# Ketogenic Diet and Gut Microbiota: Exploring New Perspectives on Cognition and Mood

**DOI:** 10.3390/foods14071215

**Published:** 2025-03-30

**Authors:** Yuhan Jiang, Yili Chen, Youmeng Chen, Xinrong Gong, Zhiyu Chen, Xin Zhang

**Affiliations:** 1 Department of Food Science and Engineering, Ningbo University, Ningbo 315211, China; 2 Ningbo Institute for Drug Control, Ningbo 315048, China

**Keywords:** ketogenic diet, gut microbiota, depression, cognitive impairment

## Abstract

The ketogenic diet (KD) is a dietary regimen characterized by low carbohydrate intake and moderate protein levels, designed to simulate a fasting state and induce ketosis for the production of ketone bodies from fat. Emerging research underscores KD’s potential in improving cognitive functions and regulating mood. Investigations into its safety and efficacy have centered on its anti-inflammatory properties and its impact on neurological health and the gut–brain axis (GBA). This review delves into the relationship between the KD and gut microbiota, emphasizing its potential role in cognitive enhancement and mood stabilization, particularly for managing mood disorders and depression. The investigation of the KD’s physiological effects and its role in promoting cognition and emotion through gut microbiota will pave the way for innovative approaches to personalized dietary interventions.

## 1. Introduction

Cognitive function encompasses various processes such as learning, memory, language, reasoning, and emotional regulation [1]. As individuals age, cognitive abilities tend to decline, and the prevalence of neurodegenerative conditions like Alzheimer’s disease (AD) and Parkinson’s disease (PD) continues to rise [2]. This trend highlights the pressing need for innovative strategies aimed at enhancing the quality of life for older adults [3]. Dietary approaches have been explored for decades, but there is growing interest in targeted nutritional interventions, such as the ketogenic diet (KD), for their potential to promote health and counteract disease progression [4].

**Figure 1:**
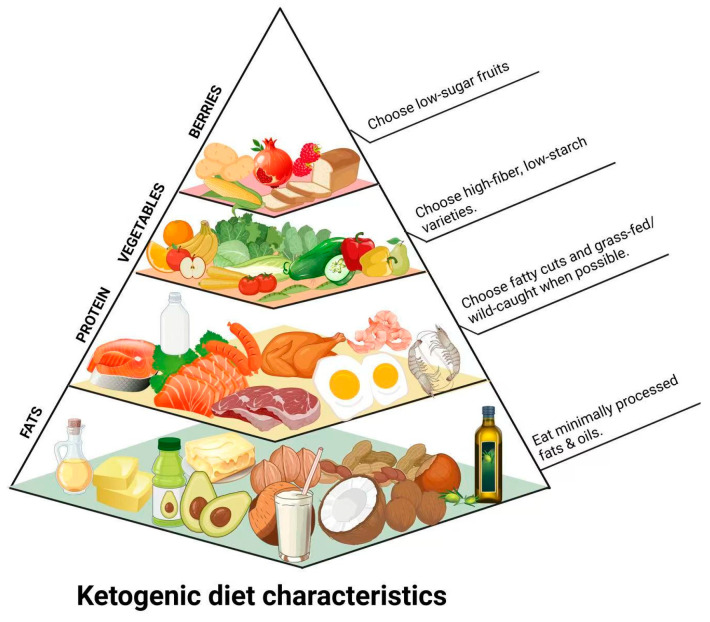
Simplified KD food pyramid illustrating food groups according to consumption frequency in a typical KD regimen, emphasizing high-fat, moderate-protein, and very low-carbohydrate food intake.

Originating in the 1920s, the high-fat, low-carbohydrate KD was initially developed as a treatment for drug-resistant epilepsy. In recent years, it has gained attention for its potential benefits in enhancing cognitive function [5]. This dietary pattern meets the body’s nutritional needs by providing ketone bodies instead of glucose for energy [6]. Ketone bodies are intermediates in the oxidative breakdown of fatty acids, including acetoacetate (AcAc), beta-hydroxybutyrate (β-HB) and acetone (Ac). KD meets nutritional requirements by strictly limiting carbohydrate intake and allowing generous consumption of fats, including saturated fats, while promoting rapid and sustained weight loss (Figure 1). Additionally, it has been associated with improvements in biomarkers, such as reduced serum hemoglobin A1c levels in patients with type 2 diabetes mellitus [7]. Furthermore, ketone therapy has been shown to enhance mitochondrial respiration, support neuronal long-term potentiation, and increase brain-derived neurotrophic factor (BDNF) expression, thereby reducing oxidative stress and inflammation [8].

The gut microbiota, often referred to as the “hidden organ” within the human body, plays a regulatory role through various mechanisms and can be adjusted or even reshaped by altering dietary habits to improve overall health [9]. Manipulating the composition of the gut microbiota has emerged as a cutting-edge strategy for enhancing health [10]. The gut–brain axis (GBA) serves as a bidirectional communication pathway between the gut and the brain, integrating metabolic functions and contributing to the development of certain diseases. This axis involves the nervous, endocrine, and immune systems [11,12]. Through this pathway, the gut microbiota influences brain function, regulates neurotransmitter production, and affects emotional responses [13].

Research has revealed a strong connection between the composition and diversity of gut microbiota and mental health, showing that an imbalance in gut microbiota is closely linked to mood disorders, including depression and anxiety [14]. An increasing number of studies reveal the role of the GBA in regulating mood and cognition, underscoring the critical role of gut microbiota in mental health. By altering the composition and metabolic activity of the gut microbiota, the KD can promote the production of short-chain fatty acids (SCFAs) and reduce gut inflammation, thereby improving cognition and alleviating depressive symptoms [15]. The KD has been shown to modulate gut microbiota, improve memory and concentration, and reduce mood swings by affecting neurotransmitter secretion. Thus, the KD offers new avenues of research and therapeutic strategies for the prevention and treatment of depression by influencing gut microbiota [16]. The primary aim of this review is to explore the complex relationship between the KD and gut microbiota, and their collective role in cognitive enhancement and mood stabilization. By integrating current findings, this paper seeks to highlight new insights into dietary interventions targeting cognition and mood.

## 2. KD and Its Mechanisms of Action on Gut Microbiota

### 2.1. Types of KD

Due to the strict requirements of the classic KD, patient compliance and tolerance are poor. Therefore, researchers have continued to improve the classical KD and have developed a common KD, which can be divided into four categories:

1. The classic KD has a fat-to-non-fat weight ratio of 4:1: Fat intake accounts for 80% of the total daily food weight (90% of energy supply), protein and carbohydrate account for 20% of the total daily food weight (10% of energy supply). It also provides sufficient vitamins and minerals and has been investigated as a potential therapeutic approach for various neurological and metabolic disorders [17,18].
2. The medium-chain triglyceride diet (MCTD) provides approximately 70% of total energy from fats, predominantly in the form of triacylglycerols containing medium-chain fatty acids [19]. MCTD has been widely used in clinical treatment due to its easy absorption, high functional rate, and good taste and has long been employed as a dietary therapy for children with refractory epilepsy, particularly those with a large appetite, the ability to tolerate a higher calorie intake, or with difficulty adhering to the strict restrictions of the classical KD [20]. Beyond its established role in epilepsy management, MCTD is currently being explored for its potential neuroprotective effects in other conditions. For instance, a study involving mice with PD demonstrated that MCTD provided neuroprotection in the substantia nigra, a brain region severely impacted by the disease. In addition to the neuroprotective effects of MCTD, there are other studies in which MCTD has been shown to be neuroprotective [21].
3. The modified Atkins diet (MAD) is a variation of KD, inspired by the widely known Atkins weight-loss diet. While it shares similar food choices with the classical KD, MAD offers greater flexibility by eliminating the need for precise macronutrient measurements. Typically, it derives around 65% of energy from fats, allowing a higher protein intake of approximately 30%. This more lenient approach to protein restriction, combined with a low carbohydrate intake, is sufficient to induce and sustain a ketosis-a metabolic state linked to the therapeutic benefits of MAD [22,23].
4. The low glycemic index diet (LGIT), the core of this type of KD, aims to achieve therapeutic effects by maintaining stable blood glucose levels. It is characterized by limiting carbohydrate intake and choosing foods with a glycemic index of less than 50 to minimize blood glucose fluctuations, and this approach has been shown to have a significant antiepileptic effect in children with intractable epilepsy [24].
5. The very low-calorie ketogenic diet (VLCKD) is a dietary regimen characterized by a significant reduction in carbohydrate intake, mimicking fasting and promoting ketone biosynthesis [25,26]. It has been increasingly utilized as a second-line intervention for obesity, particularly in patients who do not respond adequately to conventional hypocaloric diets. VLCKD has shown efficacy in improving glycometabolic profiles and restoring normal gonadal function in men with obesity [27,28].

### 2.2. Promoting the Production of SCFAs

The KD stimulates the production of SCFAs, primarily through the fermentation of dietary fiber by gut microbiota. SCFAs, including acetic acid, propionic acid, and butyric acid, serve as energy sources for intestinal epithelial cells. Beyond providing energy, SCFAs exhibit anti-inflammatory properties that help mitigate intestinal inflammation, suppress the proliferation of harmful bacteria, and enhance the abundance of beneficial bacterial populations, contributing to overall gut health [29]. After one week of treatment with the KD, children’s intestinal tracts showed a significant increase in the percentage of *Bacteroides* and *Prevotella*, which can produce SCFAs that protect the intestinal mucosa, and a significant decrease in the percentage of *Cronobacter* and *Proteobacteria* compared to the pre-treatment period [30]. Similarly, a comparative study of 14 children with refractory epilepsy and 30 healthy children revealed gut microbiota differences. Before the KD, children with epilepsy exhibited lower levels of *Bacteroides* and higher levels of toxin-producing *Cronobacter* and *Proteobacteria* compared to healthy controls. One week of KD treatment increased *Bacteroides* and *Prevotella* while reducing *Cronobacter* and *Proteobacteria* [31]. These findings highlight the KD’s potential to restore gut microbiota balance, enhance SCFAs production, and play a crucial role in promoting gut health and managing disease.

### 2.3. Regulating Gut Microbiota Composition

Diet is a major factor influencing the composition of the gut microbiota, and in recent years, studies have found that altering gut bacteria through diet and gut microbiota modifiers can affect cognitive health. A study of cognitive function and gut microbiota in AD patients suggests that an increase in *Firmicutes* and a decrease in *Bacteroidetes* are commonly associated with dysregulated microbiota profiles and negative health outcomes [16]. The KD can alter the composition and function of the gut microbiota in epileptic patients, and the abundance of *Bacteroidetes* was significantly increased in epileptic children after KD treatment, whereas the abundance of *Firmicutes* and *Actinobacteriota* was significantly decreased [32,33]. KD intervention in healthy male C57BL-6 mice resulted in a short-term decrease in bacterial abundance and diversity, with recovery observed by week 12, significantly exceeding baseline levels [34]. The abundance of beneficial microbiota such as *Akkermansia muciniphila* and *Lactobacillus* was increased, and the abundance of harmful microbiota, particularly pro-inflammatory microbiota such as *Desulfovibrio* and *Bacillus*, which have the potential to cause cancer in normal intestinal cells, was reduced [35]. It has been found that the abundance of polysaccharide-based energy-providing microbiota decreases in relative terms when carbohydrate intake is reduced, and that the abundance of this microbiota is positively proportional to the amount of carbohydrate intake, suggesting that the KD may reduce the abundance of inflammatory microbiota in the gut by reducing carbohydrate intake, thereby reducing the intestinal inflammatory response and the likelihood of cancerous transformation of normal cells in the gut [36].

Recent studies have shown that the KD, metabolized to produce ketone bodies, alters the gut microbiota by selectively inhibiting *Bifidobacteria*, thereby reducing the levels of pro-inflammatory Th17 cells in the gut [37]. Direct feeding of ketone bodies to normal chow mice also leads to similar gut microbiota changes, with alterations resembling those observed during KD intervention [38]. At the genus level, significant differences in the relative abundance of individual genera, such as *Bacteroides*, *Cronobacter*, *Erysipelothrix* and *Bifidobacterium*, were found between healthy controls and patients before the initiation of the KD. For many taxa, the differences between healthy and sick individuals are reduced by KD treatment. *Lactobacillus* and *Bifidobacterium longum* have the ability to produce SCFAs. An analysis of the association between host genetics, gut microbiota, metabolome, and memory in mice revealed that Lactobacillus in the microbiota may enhance memory by influencing neurotransmitter levels in the hippocampal region through increased production of metabolites such as lactic acid [39].

### 2.4. Affecting Intestinal Mucosa

The intestinal mucosa is an important immune barrier for the body, and when its immune function is impaired or inflammatory infiltration occurs, it can lead to various health problems. The KD has shown potential efficacy in repairing impaired immune function and reducing inflammatory infiltration in the intestinal mucosa.

#### 2.4.1. Repairing Gut Mucosal Immune Function

The intestinal barrier is critical for preventing the translocation of pathogens and maintaining immune homeostasis. The KD improves barrier integrity through multiple mechanisms, including the production of butyric acid, which promotes mucus secretion by intestinal epithelial cells. This mucus layer forms a protective barrier, isolating pathogenic bacteria and toxins from the intestinal lining [40]. Additionally, the KD enhances intestinal epithelial cell proliferation, reduces apoptosis, and maintains a robust epithelial layer, which is essential for the barrier’s structural integrity and function [41]. Despite the lack of fermentable carbohydrates, the KD maintains a robust mucus layer, which contributes to maintaining a protective intestinal mucus layer that facilitates intestinal health [42]. *Akkermansia muciniphila* is an anaerobic bacterium that colonizes the mucus layer of the human gastrointestinal tract and accounts for 1%–4% of human gut microbiota [43]. *Akkermansia muciniphila* has been found to improve metabolic function and immune response [44]. Research has shown that normal mice on a KD experience decreased blood glucose levels and body mass, elevated blood ketone levels, and improved neurovascular function. The efficacy of this treatment is closely linked to gut microbiota changes, including a reduction in harmful bacteria, such as *Desulfovibrio* and *Sutterella*, and an increase in beneficial bacteria like *Akkermansia muciniphila* and *Lactobacillus* [45]. These changes contribute to improved gut health and overall microbiota balance.

#### 2.4.2. Reducing the Extent of Inflammatory Infiltration

KD ameliorates intestinal inflammatory factors by inducing the body into a state of ketosis, and reduced carbohydrate intake decreases sugar fermentation in the gut, thereby reducing harmful metabolites that may trigger intestinal inflammation [46]. The KD increases the production of ketone bodies, and studies have demonstrated that β-HB has an anti-inflammatory effect and reduces intestinal inflammation by inhibiting NLRP3 inflammatory microsome activation [47]. The KD is rich in antioxidant components, such as certain fatty acids and ketone bodies, which reduce oxidative stress and further reduce inflammation. Studies have shown that the KD effectively reduces inflammatory marker levels in patients with inflammatory bowel disease. In a study of patients with inflammatory bowel disease, the KD significantly reduced levels of inflammatory factors and improved clinical symptoms in patients [48]. Investigation into the effects of the KD on colitis in mice revealed that the KD significantly reduced intestinal inflammatory factors and enhanced intestinal health by upregulating anti-inflammatory cytokines such as IL-10 [37].

## 3. The GBA: A New Form of Communication Between Gut Bacteria and the Brain

### 3.1. Bi-Directional Communication Between Gut Microbiota and the Central Nervous System

**Figure 2:**
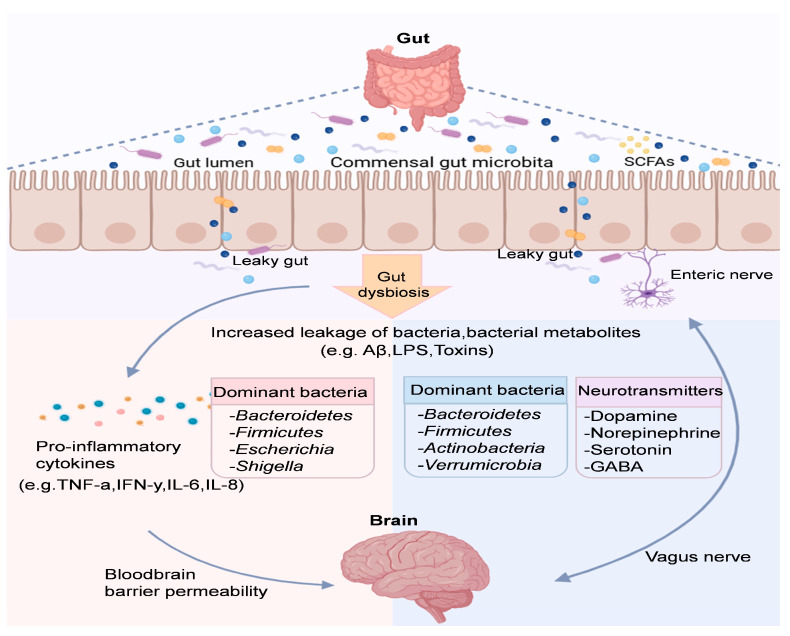
The microbiota–gut–brain axis involves pathways and molecules for communication. Neuronal circuits like the enteric nervous system and neuropods connect the gut microbiota and brain directly and indirectly.

Gut microbiota is a key regulator of the GBA and can influence brain function through the upstream gut–brain pathway [49]. Specifically, gut microbiota and their metabolites influence the enteric nervous system, and the neuroendocrine system integrates and transmits information to the central nervous system (CNS), which then processes, integrates, and responds, thereby influencing brain function and behavior [50]. The imbalance in the GBA caused by gut microbiota dysbiosis can trigger heightened inflammatory signaling and increased intestinal epithelial cell permeability. These disruptions are associated with the onset and progression of various diseases, underscoring the critical role of gut health in maintaining overall wellbeing [51]. On the other hand, the CNS also regulates the intestinal microbiota community, mucosal immunity and the immune system through the brain–intestinal pathway. Specific regions of the brain, such as the thalamus, amygdala and frontal lobe, are able to integrate relevant signals and transmit them to the enteric nervous system to regulate gastrointestinal motility and gut microbiota via the autonomic nervous system or the hypothalamic–pituitary–adrenal axis [52] (Figure 2).

### 3.2. Gut Microbiota Induces Neuroinflammation That Affects Cognition

The two-way communication of the GBA regulates gut and CNS health and mediates the neuroinflammatory response that plays a critical role in cognitive and emotional dysregulation. Just as early life development parallels the development of gut microbiota, several age-related diseases have been linked to the state of the microbiota in animals and humans. Reduced microbiota diversity has been associated with increased microglial activation, which has been linked to differences in brain mass in mice [53]. It has been reported that the neuroinflammatory mechanisms of AD are driven by the gut microbiota, which activates the human innate immune system, and that dysbiosis is an important factor in triggering neuroinflammation [54,55]. In addition, bacterial strains such as *Escherichia coli*, *Bacillus subtilis*, *Salmonella typhimurium* and *Staphylococcus aureus* can produce large amounts of functional amyloid by accumulating proteolytically misfolded Aβ oligomers and fibrils, which may be an important source of neuronal protein misfolding and thus innate immune triggering [56]. Research indicates that 73 cognitively impaired patients had increased levels of proinflammatory *Escherichia coli/Shigella*, decreased levels of anti-inflammatory rectal *Escherichia coli* in feces and peripheral blood, and decreased abundance of inflammatory complexes (NLRP3), chemokine 2 (CXCL2), and peripheral blood interleukin-1β (IL-1β) expression, suggesting that gut microbiota may drive peripheral inflammation to induce cerebral amyloidosis, leading to neurodegeneration and cognitive deficits in AD [57]. Similarly, studies in germ-free APP/PS1 mice revealed that Aβ deposition in the brain increased significantly following cecal transplants from older APP/PS1 mice. This was associated with altered astrocyte morphology and heightened inflammatory activity, further supporting the role of gut microbiota in promoting peripheral inflammation that may lead to cerebral amyloidosis and the neurodegenerative pathology characteristic of AD [58].

### 3.3. Effects of Gut Microbiota Metabolites on Cognition and Depression

Gut microbiota affects brain function and mental health through various mechanisms, and metabolism has emerged as an important pathway through which the microbiota influences depression and cognition [59]. Gut microbiota ferments dietary components such as fiber and polysaccharides, producing a variety of key metabolites, including SCFAs, neurotransmitters, and bacterial lipopolysaccharides [60]. These metabolites not only act locally in the gut, but also influence the CNS via the bloodstream, resulting in direct or indirect effects on mood and cognitive regulation. Butyric acid, one of the most important components of SCFAs, is a major energy source for colonic epithelial cells and plays a key role in maintaining intestinal health [61]. Over 95% of butyric acid is produced and absorbed in the colon, where it protects against cancer by maintaining the stability of colonocytes, regulates the balance of intestinal microbiota, and is effective in improving conditions such as irritable bowel syndrome and antibiotic-associated enteritis. Notably, butyric acid not only supports intestinal epithelial cells, but can also cross the blood–brain barrier (BBB) into the CNS, where it exerts anti-inflammatory and neuroprotective effects [62]. This direct action contributes to improved cognitive function and mood stabilization, demonstrating the important value of SCFAs outside the gut [63]. A study comparing the composition of the distal gut microbiota of 70 healthy and 101 depressed children found that fecal samples from children with depression lacked a wide range of SCFAs-producing bacteria, including those from the genera *Subdoligrinum*, *Dialister*, *Fuscatenibacter*, *Ruminococcus* and *Dorea* [64]. SCFAs play a critical role in maintaining the homeostasis of the regulatory T cell population, and their absence weakens intestinal immune function, leading to the entry of intestinal bacteria and toxins into the bloodstream through a leaky intestinal wall. This, in turn, activates the systemic immune system and induces aberrant behavioral and emotional changes. In addition, in studies of adolescent depression, sertraline treatment has been found to restore the abundance of strains such as *Faecalibacterium* and *Ruminococcus* and improve depressive behavior by promoting the expression of tryptophan hydroxylase 1 or 2 and by increasing levels of 5-hydroxytryptamine (5-HT) in the brain and colon. Supplementation with *Ruminococcus* protects against synapse loss and maintains microglia and astrocyte homeostasis in depressed mice [65]. Therefore, modulating gut microbiota and their metabolites has emerged as a promising pathway to improve mood and cognitive function.

## 4. Potential Mechanisms Underlying the Neuroprotective Effects of KD

### 4.1. Neuroprotective Effects of KD

#### 4.1.1. Neurotransmitter Pathways

The KD modulates neurotransmitter levels in the brain while exerting systemic effects via the GBA. By enhancing the production of glutamate and gamma-aminobutyric acid and reducing glutamate-induced excitotoxicity, the KD helps attenuate neuronal damage, especially in diseases like epilepsy [66]. Additionally, the KD influences serotonin 5-HT levels through interactions with the gut microbiota, which may aid in stabilizing mood and alleviating symptoms of anxiety and depression. In animal studies, acetate was shown to cross the BBB and regulate neurotransmitter levels in the hypothalamus. Moreover, propionic acid promoted the expression of tryptophan hydroxylase 1, a key enzyme in serotonin synthesis, thereby enhancing 5-HT production [67].

**Figure 3:**
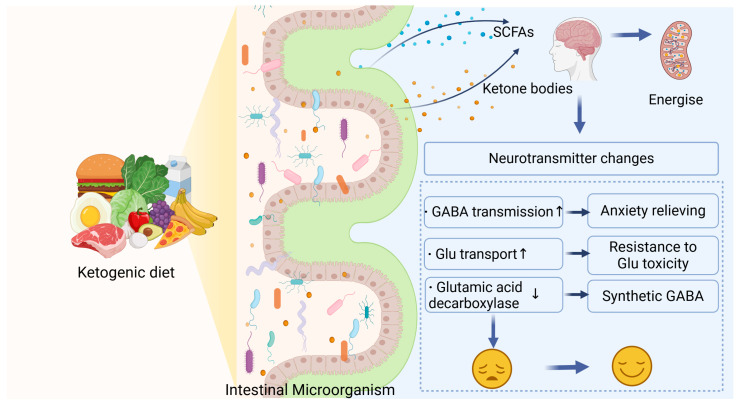
The KD modulates gut microbiota, increasing the production of ketone bodies and SCFAs. These metabolites enter the brain, regulate energy metabolism and neurotransmitter levels, enhance GABA transmission and glutamate transport, and reduce glutamic acid decarboxylase expression, contributing to mood improvement.

Pavon’s research concluded that KD-induced ketosis shifts energy metabolism from glycolysis to ketone body utilization, a shift that protects against oxidative stress, neuroinflammation, and impaired insulin sensitivity. These mechanisms together stabilize brain function and are expected to attenuate cognitive decline in neurodegenerative diseases [68,69]. The overlap between the KD’s pathway and the effects of functional foods has spurred interest in developing food-based interventions, such as MCT-enriched products and probiotics, that can mimic or amplify the benefits of the KD [70,71,72] (Figure 3).

#### 4.1.2. Anti-Inflammatory and Immunomodulatory Effects

The KD has garnered attention for its antioxidant and anti-inflammatory properties, with β-HB being central to these effects [73]. Chronic inflammation and oxidative imbalance are strongly linked to mood disorders like depression and neurodegenerative diseases. For instance, major depression often involves persistent inflammation [74]. Evidence suggests that β-HB modulates the NLRP3 inflammasomes, which are involved in the worsening of neurodegenerative diseases such as AD [75].

In AD, amyloid plaques activate microglia, triggering NLRP3 inflammasomes and releasing pro-inflammatory mediators [76,77]. Research has shown that β-HB inhibits this cascade by activating HCAR2 receptors, thereby preventing microglia activation and reducing inflammatory cytokines like IL-1β and TNF-α [78]. The potential of the KD in alleviating depressive symptoms is also supported by clinical studies indicating improved mood in depressed patients [79,80]. Furthermore, the KD’s mood-stabilizing effects in bipolar disorder have been explored, as it may help regulate mood swings by reducing oxidative stress and stabilizing neuronal function [81].

#### 4.1.3. Antioxidant Effects

Impaired glucose utilization and mitochondrial dysfunction are key factors in the progression of dementia [82]. The imbalance in energy metabolism, due to reduced glucose uptake and inefficient glycolysis, impairs the processing of amyloid precursor proteins, leading to the generation of neurotoxic amyloid peptides. The KD has shown antioxidant properties that can reduce oxidative stress and neuronal damage by minimizing reactive oxygen species production [83,84]. Animal models of AD support these findings, showing reductions in Aβ peptide levels, improved mitochondrial function, and reduced tau protein pathology [85].

At the molecular level, the KD regulates antioxidant pathways such as the Nrf2 signaling pathway, which controls the expression of antioxidant proteins to combat oxidative damage [86]. The KD activates Nrf2, significantly reducing acute oxidative stress after just three weeks of intervention [87,88]. Additionally, the stress protein heme oxygenase-1 is involved in the KD’s antioxidant effects, promoting neuroprotection in motor neurons [89,90].

### 4.2. KD and Gut Microbiota Interact to Modulate Cognitive Impairment

The KD’s neuroprotective effects extend to the modulation of the gut microbiota, a key player in cognitive impairment and neurodegenerative diseases [91]. The KD alters the gut microbiota composition, promoting the production of beneficial metabolites, such as SCFAs and ketone bodies, which cross the BBB to reduce neuronal damage and improve synaptic function. In addition to its direct neuroprotective effects, KD-induced changes in the gut microbiota modulate systemic inflammation, reducing pro-inflammatory signals and enhancing anti-inflammatory responses to improve the brain microenvironment. These changes are closely linked to improved BBB function and reduced accumulation of neurotoxic substances in models of AD and mild cognitive impairment (MCI) [92].

Although other dietary patterns, such as the modified Mediterranean diet, also show potential in enhancing cognitive function, they primarily affect the microbiota through fiber and polyphenols, with limited effects on ketosis and β-HB levels [93]. Therefore, while the MMD improves cognitive function, it lacks the mechanisms to enhance BBB function and reduce inflammation to the same degree as the KD. The KD’s ability to induce ketosis, elevate β-HB levels, and improve brain health through these mechanisms provides a comprehensive strategy for neuroprotection, particularly in diseases like AD and MCI [94].

## 5. Conclusions

Taken together, by altering the gut microbiota and its metabolites, the KD may have a positive role in maintaining normal cognitive function and regulating mood, especially in the treatment of neurological disorders and depression. While the KD has shown promise in improving cognition and mood, its therapeutic application faces several limitations, such as challenges in long-term adherence due to dietary restrictiveness, potential nutrient deficiencies (e.g., fiber, vitamins, and minerals), variability in individual responses, and insufficient long-term clinical evidence. Therefore, further research is needed to better elucidate the complex relationship between the KD, gut microbiota, and neurological health to design individualized KD protocols, identify the optimal timing and duration of interventions, and develop personalized KD strategies effective in preventing or slowing cognitive decline and mood disorders.

## Data Availability

The original contributions presented in the study are included in the article, further inquiries can be directed to the corresponding author.

## Abbreviations

The following abbreviations are used in this manuscript:

KD	Ketogenic diet
GBA	Gut–brain axis
AD	Alzheimer’s disease
PD	Parkinson’s disease
β-HB	Beta-hydroxybutyrate
BDNF	Brain-derived neurotrophic factor
SCFAs	Short-chain fatty acids
MCTD	Medium-chain triglyceride diet
MAD	The modified Atkins diet
LGIT	Low glycemic index diet
CNS	Central nervous system
BBB	Blood–brain barrier
5-HT	5-hydroxytryptamine
MCI	Mild cognitive impairment
